# A randomized assessment of the impact of ‘Those Nerdy Girls’ newsletters on adult vaccination outcomes

**DOI:** 10.1371/journal.pone.0344258

**Published:** 2026-03-12

**Authors:** Malia Jones, Megan Reilly, Amanda Simanek, Alexis Stewart, Roopa Seshadri, Jennifer B. Dowd, Ashley Z. Ritter

**Affiliations:** 1 Department of Community and Environmental Sociology, University of Wisconsin-Madison, Madison, Wisconsin, United States of America; 2 School of Medicine and Public Health, University of Wisconsin-Madison, Madison, Wisconsin, United States of America; 3 Department of Foundational Sciences and Humanities, Michael Reese Foundation Center for Health Equity Research, Chicago Medical School, Rosalind Franklin University of Medicine and Science, North Chicago, Illinois, United States of America; 4 Department of Sociology, University of Wisconsin-Madison, Madison, Wisconsin, United States of America; 5 Magnolia Impact Solutions, Philadelphia, Pennsylvania, United States of America; 6 Nuffield Department of Population Health, Leverhulme Centre for Demographic Science, University of Oxford, Oxford, United Kingdom; 7 Hunter-Bellevue School of Nursing, Hunter College, City University of New York, New York, New York, United States of America; McGill University, CANADA

## Abstract

Adult vaccine-preventable infectious diseases contribute substantial burden each year, and vaccine uptake remains suboptimal. As digital health communication grows, digital newsletters may represent a scalable, low-cost tool to promote vaccination. We conducted a randomized prospective study to evaluate the impact of digital newsletters on subscribers’ knowledge, attitudes, and behaviors regarding four adult vaccines: respiratory syncytial virus (RSV), influenza, shingles, and COVID-19. Between November 2023 and January 2024, half of subscribers to the *Those Nerdy Girls* (TNG) Substack newsletter received additional vaccine-focused newsletters, while the other half received only the standard twice-weekly newsletters. Pre- and post-intervention online surveys assessed vaccine knowledge, attitudes, and behaviors among adult subscribers who consented to participate. Across 1,327 pre-intervention and 1,208 post-intervention participants, knowledge gains were observed for RSV and shingles vaccines in the intervention group compared with controls, while knowledge of influenza and COVID-19 vaccines did not improve. Attitudes toward vaccination were generally positive at baseline and showed no significant intervention effects across vaccine types. Likelihood of vaccination increased for influenza, COVID-19, and RSV overall during the study period, but only influenza vaccination showed a significantly greater increase in the intervention group relative to controls. No significant effects were found for shingles vaccination. These results indicated that digital newsletters can improve knowledge and, in some contexts, support uptake, but knowledge gains did not consistently translate into changes in attitudes or behaviors. Findings highlighted the limitations of knowledge-deficit models for adult vaccination promotion. Digital newsletters should be considered as one component of broader public health strategies, particularly when paired with approaches that address logistical access, trust, and social norms.

## Introduction

At least 50,000 U.S. adults are estimated to die each year of vaccine-preventable diseases, with tens of thousands more experiencing hospitalization and millions more who become ill [1]. Common, vaccine-preventable adult illnesses include influenza, COVID-19, respiratory syncytial virus (RSV), and shingles [2,3]. Yet, adult vaccine uptake remains relatively low [3]. The Centers for Disease Control and Prevention (CDC) estimated that there were 9–41 million influenza cases, 120,000–710,000 hospitalizations, and between 6,300–52,000 deaths annually between 2010–2024 [4]. Despite influenza’s burden, from 2010–2024, only 37.1 to 50.2% of U.S. adults received an annual influenza vaccine [5].

Uptake among U.S. adults has been even lower for vaccines that have more recently become available. While 34,000−53,000 deaths and 290,000−450,000 hospitalizations from COVID-19 occurred from October 2024-July-2025 [6], only 23% of U.S. adults received the updated 2024−25 COVID-19 vaccine [7]. Indeed, influenza vaccine rates for adults have declined since the onset of the COVID-19 pandemic [5], in part due to spillover of COVID-19 vaccine hesitancy [8].

On July 21 2023, the Centers for Disease Control and Prevention (CDC) Advisory Committee on Immunization Practices (ACIP) recommended that people aged 60 years and older *may* receive a single dose of a vaccine against RSV, based on shared clinical decision-making [9]. This recommendation was revised the following year (after the study described here was concluded) [10]. Approximately 2–10% of older adults in the U.S. get RSV each year, leading to an estimated 11,000 annual deaths [11]. While one dose of the adult RSV vaccine is 75% effective at preventing RSV-related hospitalization [12], only 47.5% of U.S. adults aged 75 years and older received this vaccine as of April 2025 [7].

Beginning in 2017, ACIP recommended two doses of the shingles vaccine for all adults aged at least 50 years, and those aged 19 years and older with impaired immune systems [13]. Current estimates suggest that 1 in 3 people will develop shingles in their lifetime, and 10–18% of those who do will develop long-term nerve pain (i.e., postherpetic neuralgia) [14]. While two doses of shingles vaccine is 90% effective at preventing shingles and postherpetic neuralgia, as of 2018 fewer than 35% of U.S. adults aged 60 years and older were vaccinated [15].

In an increasingly digital health information environment [16], a growing number of interventions have focused on the rapidly evolving role that digital communication tactics may play in improving vaccination uptake among adults [17]. Such tactics include reminder phone calls, texts, and emails, often from clinicians or government health agencies; informational emails from official sources (such as clinicians or health systems); and health information from informal sources using modalities such as subscription-based digital newsletters, blogs, and social media accounts [16,18].

### Automated digital reminders

Automated digital reminders in health communications refer to prompts delivered via digital channels (e.g., email, SMS/text messages, app notifications) that cue individuals to perform a health-related behavior [19]. These interventions attempt to nudge users toward desired health actions outside of direct clinical encounters [19]. Such digital reminders have been leveraged to cue those who are vaccine-eligible to get vaccinated [20,21]. However, evidence of the effectiveness of such reminders on increasing vaccine uptake has been mixed [22,23]. One recent systematic review focused on older adults found that, across digital reminder modalities (e.g., emails, texts, etc.), more than half of reviewed studies reported some evidence of increased vaccination uptake, though effects were heterogenous and often modest [24]. Another systematic review concluded that email reminders about vaccination were generally more effective than no intervention, but tended to be less effective or no more effective than other automated reminder modalities (e.g., texts, app notifications, phone calls), with variation across populations and study designs [25].

### Informational communications

Digital reminders typically contain only a prompt to action, not information or educational content (e.g., descriptions of diseases, vaccines, eligibility criteria, or benefits and risks). Some recent vaccine promotion efforts have also focused on sending emails containing vaccine-related information. Such efforts are consistent with the knowledge-attitude-behavior theory of change, which suggests that providing information on vaccines will translate to changes in attitudes and subsequently vaccine behavior, given the information comes from trusted sources [26]. This framework aligns with the Health Belief Model, in which increasing knowledge can influence perceived key determinants of individuals’ motivation to engage in preventive health behaviors like vaccination, such as susceptibility, severity, benefits, and barriers [27].

A cluster-randomized trial conducted in Denmark directly compared the effect of reminders and informational content, both sent via email, from the Danish Health Authority. The informational email contained facts on influenza and COVID-19 vaccines [28]. The authors found that, compared to the simple reminder, repeated informational emails sent 14 days apart were associated with greater influenza vaccine uptake [28]. These findings suggest that email-based dissemination of vaccine-related information by a government health authority has a positive impact on influenza vaccine uptake among adults. Questions remain, however, regarding the impact of informational digital content on uptake of other adult vaccines.

People are also increasingly seeking health information online from less formal health authorities, such as content from social media platforms, YouTube creators, digital newsletters, and blogs [29]. In many of these modalities, users self-select or “subscribe” to content that interests them. Little is known about how vaccine-related information disseminated from such sources may affect changes in vaccine knowledge, attitudes, and behaviors [30,31]. One recent study assessed the effect of health information content shared a professional science communicator across a variety of digital media modes on adult influenza vaccine knowledge, attitudes, and behaviors [32]. Content in this study included digital newsletters delivered via a self-subscribing system, as well as podcasts, social media posts, and infographics. Results from cross-sectional surveys administered before and after the campaign showed that 4.7% of post-campaign respondents reported that they changed their minds about getting vaccinated for flu; a little more than half of those said the change was in part because of the targeted educational content of the campaign [32]. Given that the informational content was shared across a range of digital platforms and mediums, it is challenging to discern, however, which aspect(s) of the campaign most influenced the changes observed. In addition, only the adult influenza vaccine was studied. Taken together, there is a need to further assess the impact of digital communications that include informational content beyond vaccine reminders and are sent from entities other than clinicians and government agencies, on vaccine knowledge, attitudes, and behaviors for a wider array of adult vaccines. Our intervention is situated at the intersection of these emerging communication strategies.

### Prospective randomized impact assessment of informational digital newsletter content on adult vaccine behaviors

Those Nerdy Girls (TNG), a collective of women and nonbinary scientists and clinicians, have provided practical, fact-based health information via multiple social media platforms since March 2020 and Substack digital newsletters since July 2021 [33–37]. Substack is a popular digital newsletter aggregator and delivery service that allows users to self-subscribe to content that interests them. Content on Substack is delivered to subscribers’ email inboxes and can also be viewed on the Substack app or website. Substack also suggests content that may be of interest to users based on its internal algorithms. TNG’s content across all platforms (including the Substack newsletter) includes practical information about COVID-19 and other infectious disease prevention, pediatric and adult vaccinations, reproductive health, mental health, aging, and data literacy.

In this study, we used a prospective, randomized design to measure the impact of an informational digital newsletter, delivered via Substack to an existing subscriber list, on vaccine knowledge, attitudes, and behaviors for four adult vaccines: RSV, influenza, shingles, and the Fall 2023 season COVID-19 vaccine. Between November 2023 and January 2024, half of subscribers to the TNG Substack newsletter received additional vaccine-focused newsletters, while the other half received only the standard twice-weekly newsletters. We hypothesized that following receipt of additional digital newsletters, vaccination knowledge, attitudes, and vaccine uptake for these four vaccines would increase more in the intervention group compared to the control group.

## Methods

### Intervention

**Fig 1:**
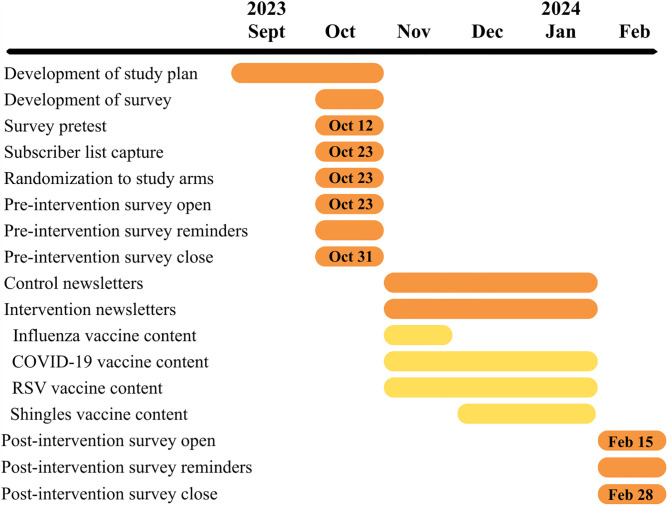
Timeline of study procedures.

At the time of the study launch (October 23, 2023), TNG’s free Substack newsletter had 12,721 subscribers. TNG typically sends its Substack subscribers twice-weekly newsletters, which are delivered via email and/or in the Substack app. We randomly assigned all TNG Substack newsletter subscribers to one of two groups. Half of the subscribers were assigned to a control group and received TNG’s regularly scheduled newsletters twice a week throughout the study. The other half were assigned to the intervention group, which received the same two newsletters *plus* two additional TNG newsletters each week featuring additional content about four adult vaccinations: RSV, shingles, influenza, and the Fall 2023 COVID-19 vaccine. A timeline of our study procedures is shown in Fig 1.

**Fig 2:**
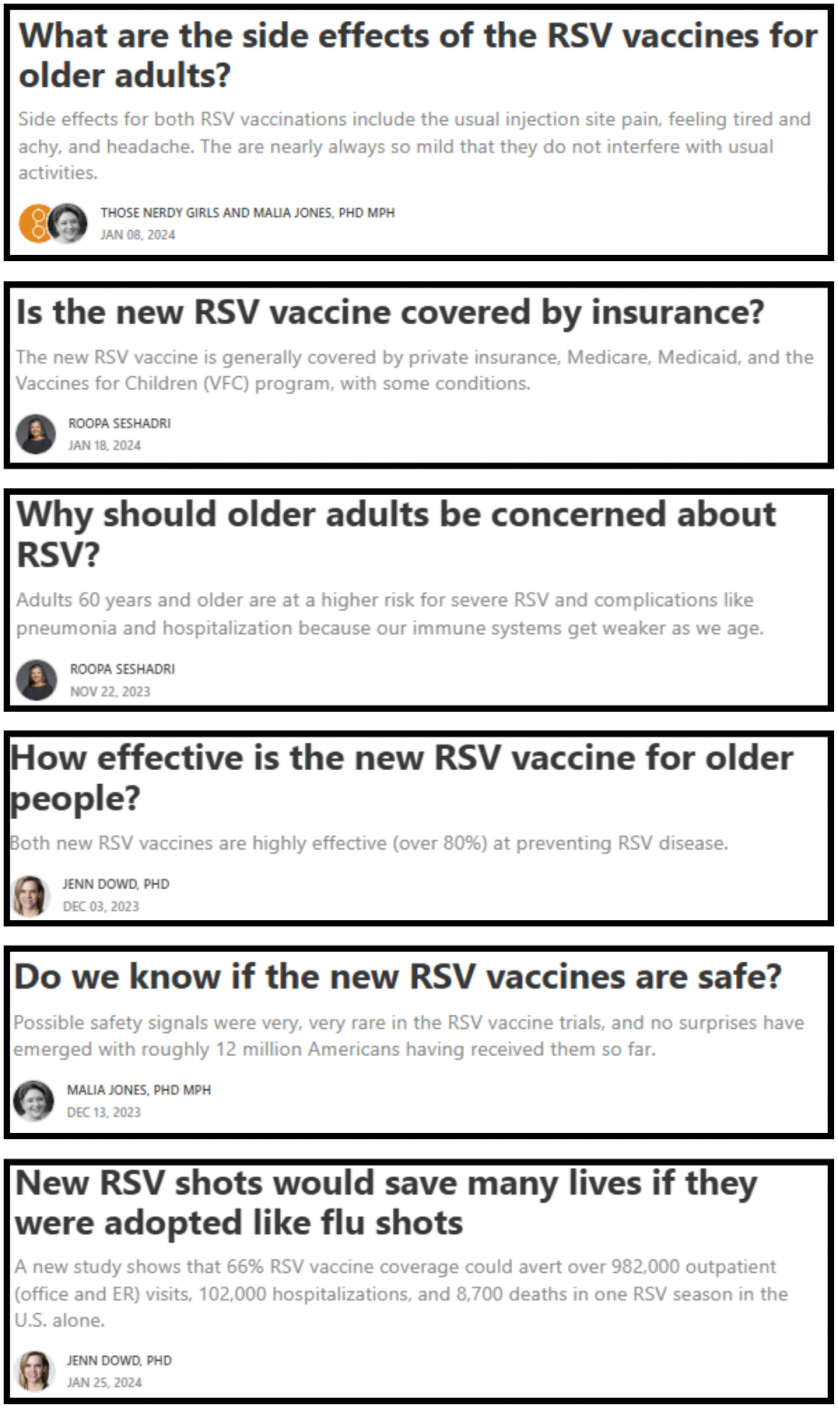
Example intervention content: Digital newsletters about adult RSV vaccine.

In total, intervention group subscribers received an additional 24 digital newsletters between November 1, 2023 and January 31, 2024. Each newsletter focused on a single vaccine. Content was distributed such that each of the four vaccines was featured in six separate newsletters over the intervention period (4 vaccines x 6 newsletters = 24 total). Because the intervention occurred during influenza vaccination season, which is relatively brief, content on influenza vaccine was concentrated during the first month of the study period (beginning Nov 1, 2023), and content on the other vaccines was distributed across the remaining 8 weeks. The full content, timing, and open rates of the individual intervention digital newsletters are available in S1 Appendix.. Example content is shown in Fig 2.

All digital newsletters followed the format of regular TNG Substack content. That is, they consisted of 300–600-word essays, and included a title and a leading short summary or Single Overarching Communication Objective [38] statement (examples in Fig 2). Intervention newsletters were written by TNG contributors with expertise in epidemiology, population health, and health policy. The intervention group newsletters provided information on topics including vaccine side effects, need for an updated vaccine where relevant, vaccine recommendation details (e.g., recommended ages and doses), vaccine efficacy, vaccine safety, morbidity and mortality from each virus, and cost of vaccines. Additional descriptive information about the newsletter topics is available in S1 Appendix.

### Pre- and post-intervention survey and sample

Prior to the launch of intervention content (on Oct 23, 2023) and following the conclusion of the intervention (on February 15, 2024), all TNG Substack subscribers were invited via email to complete an online survey assessing knowledge, attitudes, and behaviors around the target vaccinations. Demographic information was also collected on either the pre- or post-intervention surveys, including gender, educational attainment, income quartile, household size, health insurance status, rural-urban self-identification, and political orientation (see response options in S2 Appendix). Age was collected at both times. The survey was fielded using Qualtrics software [39]. Eligible participants were aged at least 18 years and living in the U.S. TNG Substack newsletter subscribers received up to three email reminders to complete each survey. TNG also posted notices that each survey was open on its social media pages. There was no incentive to participate. The pre-intervention survey remained open for one week, and the post-intervention survey was open for two weeks.

During the study period, we received 683 requests to unsubscribe from TNG content via the Substack platform. Unsubscribe requests were tracked and synced to the survey invitation list to prevent those who unsubscribed from being invited to complete the post-intervention survey. Unsubscribe requests were evenly distributed between the control and intervention groups. Unsubscribe requests during the study period were also similar to the typical rate of unsubscribe requests TNG receives, about 1.5% per month.

**Fig 3:**
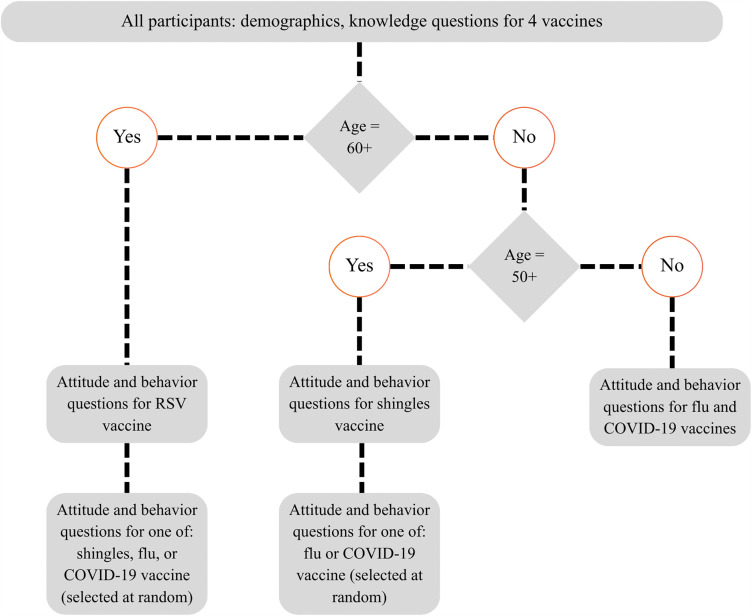
Visualization of age and random assignment in pre- and post-intervention surveys.

At both pre- and post-intervention phases, all participants answered questions about their knowledge of all four vaccinations (whether they were age-eligible for the vaccination or not). In addition, each participant answered questions about their attitudes and behaviors for two vaccinations, depending on their age and/or random assignment, using a workflow illustrated in Fig 3.

We used differentiation by age to assign attitude and behavior questions because age-specific recommendations for each vaccination varied. At the time of the study, all people aged at least 60 were recommended to discuss RSV vaccination with a provider [9]; all people at least 50 were recommended to receive a two-dose shingles vaccination every 10 years [13]; and all adults were recommended to receive both influenza and COVID-19 vaccination each year [40]. Therefore, participants aged at least 60 years (at the time of each survey) received questions about RSV vaccination plus one of the other three vaccines, selected at random. Participants aged between 50 and 60 years received questions about shingles vaccination plus either the COVID-19 or influenza vaccine, selected at random. Participants aged younger than 50 years received questions about COVID-19 and influenza vaccination. Additional survey domains related to vaccination information-sharing behavior, diffusion of innovation, and subjective norms were also assessed but are not analyzed here. The full survey instrument is listed in S2 Appendix. The Minimum Risk IRB of the University of Wisconsin-Madison oversaw this study (protocol #2023−1332). Study participants gave written informed consent prior to participation.

### Measures

#### Knowledge measures.

Study participants answered 6 knowledge questions about each vaccine. Response options included true, false, and “not sure.” For each vaccine, we created a vaccine knowledge index (i.e., count of incorrect answers; higher scores indicate lower knowledge) by summing the incorrect and “not sure” responses. Items were reverse coded as appropriate. To align with common reporting conventions, we computed Cronbach’s alpha separately for knowledge about each vaccine, which was low to moderate (influenza = 0.13, COVID-19 = 0.39, shingles = 0.46, RSV = 0.51). Because the knowledge assessments were designed as brief factual quizzes rather than psychometric scales intended to measure an underlying construct, internal consistency among items was not expected, and a low alpha is not necessarily indicative of poor measure quality in this context.

#### Attitude measures.

We based the attitude measures on the 5 C’s psychological antecedents of vaccine hesitancy [41], which include 5 subdomains of vaccine hesitancy: confidence, complacency, constraint, calculation, and collective responsibility. Each subdomain was assessed with multiple items, and participants rated their attitudes on a Likert scale, from strongly disagree (1) to strongly agree (5). There were 28 items assessing attitudes for shingles and RSV vaccines and 29 total items for influenza and COVID-19 vaccines. Items were reverse coded as appropriate. An overall vaccine attitude measure was constructed by calculating the mean across all items; higher scores indicate more positive attitudes. We computed Cronbach’s alpha for vaccine attitudes, which were high (influenza = 0.81, COVID-19 = 0.83, shingles = 0.82, and RSV = 0.77).

#### Behavior measures.

For each vaccine, the survey included questions assessing whether the respondent was up to date on the vaccine, and if not, whether they had intentions to get the vaccine in the next 30 days. These terms, and the questions used to assess them, varied slightly by vaccine to capture the nuances of vaccine recommendations (see S2 Appendix). For influenza and COVID-19 vaccines, we assessed whether the participant was up to date by asking if the participant had received a vaccine *this season* (example question text: “Since July 1st, 2023, have you received a flu vaccine? (That’s this season.)” Response options were “Yes,” “No,” and “Not sure.”) To assess whether participants were up to date on shingles vaccine recommendations, we asked if the participant had ever received *any* shingles vaccine. Those who answered yes were asked if it was a one-dose (Zostavax) vaccine or a two-dose (Shingrix) vaccine, and approximately when they had received it. We combined these responses analytically determine whether the participant was up to date with the current shingles vaccine recommendation. For RSV, the recommendation at the time was to talk to a provider about RSV vaccination. We therefore asked participants if they had *talked to a clinician* about RSV vaccination, what that recommendation was if so, and if they had received an RSV vaccine. In all cases, “not sure” answers were combined with “no” answers for analysis. Each behavioral outcome is measured with a single binary variable.

### Analysis

All analyses were performed using Stata version 19 [42]. For analysis, we excluded all respondents who did not qualify for the study because they were not at least 18 years old (pre-intervention n = 4; post-intervention n = 1), were living outside the U.S. (pre-intervention n = 136, post-intervention n = 54), and those with less than 5% survey completion (pre-intervention n = 425, post-intervention n = 354).

#### Descriptive analysis.

We evaluated balance in demographic characteristics across intervention and control groups at pre- and post-intervention using Pearson chi-squared tests for independence. Demographic variables included age, gender, education level, income, household size, health insurance status, rural-urban classification, and political orientation.

#### Outcome models.

The analysis followed a pre-post design with a control group comparison. The main outcomes (vaccine knowledge, attitudes, and behaviors) were examined across four vaccine types (RSV, COVID-19, influenza, and shingles) using a regression modeling framework. For each outcome, we estimated models including independent variables for time (pre/post), group (intervention/control), and their interaction. The primary purpose of regression modeling is to assess the statistical significance of difference-in-differences. We used regression modeling approaches appropriate to the distribution of each outcome variable. Specifically, vaccine knowledge (count variables with strong right skewness) were modeled using Poisson regression. The vaccine attitude scales (continuous variables) were modeled using linear regression. For modeling, we transformed the vaccine attitude scale by z-standardizing to improve normality of its distribution. Behavior outcomes (binary variables) were analyzed using logistic regression.

To maintain consistency in analytic samples across knowledge, attitude, and behavioral outcomes for each vaccine, results for vaccine knowledge were restricted to the subset of participants who are age-eligible for the relevant vaccine. We reported results from unadjusted models. We conducted sensitivity analyses (not reported) in which we ran models for all outcomes adjusted for demographic variables (age, gender, educational attainment, income level, household size, political orientation, and rurality) to assess the potential impact of residual confounding on our results. Last, we also carried out subset analyses (not reported) in which we ran models limiting the analytic sample to repeat participants, using a repeated-measures (longitudinal) design.

## Results

### Sample characteristics

Open rates on intervention newsletters ranged from 48–59%, depending on the specific message (see S1 Appendix). Table 1 presents the number of participants by group and survey round. Completed survey response rates were approximately 10% in both the intervention and control groups, consistent across both pre-intervention and post-intervention. Demographic characteristics of the sample (Table 1) reflect the broader readership of TNG (i.e., across all platforms), based on metadata from TNG’s social media accounts (data not published). The modal respondent in this sample was a white woman aged 60–69 years with a graduate degree. Most participants reported progressive political views. Pearson’s chi-squared tests with a threshold of p < 0.05 indicated that all demographics were balanced across intervention and control groups at both pre- and post-intervention. Demographic characteristics were also not statistically different between the overall pre- and post-intervention samples.

**Table 1:** Sample description.

	Pre-Intervention		Post-intervention	
	Control	Intervention	Control	Intervention
Age				
18-29	1%	<1%	1%	1%
30-39	9%	8%	8%	8%
40-49	22%	23%	23%	25%
50-59	20%	19%	22%	22%
60-69	29%	29%	28%	26%
70+	20%	20%	18%	19%
No response	0%	0%	<1%	0%
Gender				
Woman	89%	91%	88%	87%
Man	10%	7%	10%	11%
All Others	1%	1%	1%	1%
No response	<1%	<1%	<1%	<1%
Race				
White	92%	92%	91%	91%
Asian	2%	2%	2%	1%
Latin@	1%	1%	1%	2%
Black	<1%	1%	1%	1%
Multiple	3%	3%	3%	4%
All Others	1%	<1%	1%	<1%
No response	<1%	1%	1%	1%
Income				
< $30k	3%	3%	2%	3%
$30-70k	13%	15%	16%	14%
$70-120k	31%	28%	30%	27%
> $120k	40%	42%	39%	42%
No response	13%	12%	14%	14%
Education				
< 4-year degree	9%	9%	11%	11%
4-year degree	30%	34%	30%	29%
Master degree	41%	37%	37%	36%
Terminal degree	17%	18%	20%	22%
No response	2%	2%	2%	2%
Health Insurance				
Private	61%	60%	64%	63%
Public	27%	24%	24%	23%
Multiple	9%	12%	8%	10%
All Others	3%	3%	3%	3%
None	1%	<1%	1%	<1%
No response	<1%	<1%	<1%	<1%
Politics				
Progressive	79%	84%	81%	82%
Moderate	11%	9%	11%	11%
Conservative	9%	6%	7%	6%
No response	1%	1%	1%	1%
Urbanicity				
Metro	90%	88%	89%	89%
Non-metro	10%	11%	10%	10%
No response	<1%	<1%	1%	<1%
Sample Size (n)	671	656	582	624

### Knowledge outcomes

Incorrect answers to knowledge questions were rare at pre-intervention across all vaccines, with a mean between 0.76 and 1.54 knowledge items incorrect (out of 6) (Table 2, Panel A). At post-intervention, participants got fewer knowledge items wrong across all vaccines (0.68–1.57 items incorrect).

**Table 2:** Mean (SD) of outcomes at pre-intervention and post-intervention, by group.

Panel A. Knowledge Scores (Count of Incorrect Responses out of 6 Possible)						
	Pre-intervention			Post-intervention		
	Control	Intervention	n	Control	Intervention	n
Influenza	1.48 (1.01)	1.47 (1.04)	1,327	1.57 (1.04)	1.47 (1.06)	1,206
COVID-19	0.77 (0.93)	0.76 (0.91)	1,327	0.77 (0.92)	0.68 (0.89)	1,206
RSV	1.30 (1.30)	1.50 (1.37)	646	1.29 (1.23)	1.07 (1.08)	545
Shingles	1.52 (1.18)	1.54 (1.18)	900	1.54 (1.17)	1.30 (1.06)	808
**Panel B. Attitude Scores** Scores are z-standardized to the vaccination-specific distribution across both groups and time points.						
	**Pre-intervention**			**Post-intervention**		
	**Control**	**Intervention**	**n**	**Control**	**Intervention**	**n**
Influenza	−0.03 (1.00)	−0.03 (1.00)	710	0.03 (1.04)	0.03 (0.95)	649
COVID-19	−0.04 (0.99)	0.01 (0.95)	728	0.01 (1.01)	0.02 (1.05)	694
RSV	−0.29 (0.88)	−0.31 (0.92)	635	0.27 (1.02)	0.44 (0.97)	536
Shingles	−0.01 (0.97)	−0.10 (1.02)	474	0.04 (0.99)	0.08 (1.02)	432
**Panel C. Vaccination Rates**						
	**Pre-intervention**			**Post-intervention**		
	**Control**	**Intervention**	**n**	**Control**	**Intervention**	**n**
Influenza	75% (43%)	69% (46%)	712	86% (35%)	91% (29%)	436
COVID-19	61% (49%)	58% (49%)	730	75% (43%)	77% (42%)	501
RSV clinical consult	51% (50%)	51% (50%)	636	56% (50%)	59% (49%)	457
RSV vaccination	26% (44%)	30% (46%)	637	46% (50%)	51% (50%)	457
Shingles	60% (49%)	65% (48%)	474	54% (50%)	56% (50%)	318

Results of Poisson regression models with vaccine knowledge as the outcome of interest are shown in Table 3. The interaction terms in these models show the difference-in-differences (comparing pre-/post-intervention differences between the control and intervention groups). This term was statistically significant at p < 0.05 for RSV and shingles vaccine knowledge, demonstrating improved knowledge to a greater extent in the intervention group. Specifically, for RSV vaccine, the intervention respondents had a 28% greater decline in the rate of incorrect responses to knowledge questions relative to the control group (IRR of the interaction term = 0.72, CI 0.59–0.88). For shingles vaccine, the intervention group had a 16% greater decline in the rate of incorrect responses (IRR of the interaction term = 0.84, CI 0.71–0.98). However, there was no significant difference-in-difference for either influenza or COVID-19 vaccine knowledge.

**Table 3:** Difference-in-differences poisson regression results for vaccine knowledge outcomes: incidence rate ratios (CIs) for incorrect responses.

	Influenza	COVID-19	RSV	Shingles
Round				
Pre-intervention	Ref	Ref	Ref	Ref
Post-intervention	1.06 (0.97–1.15)	0.99 (0.87–1.12)	0.99 (0.86–1.15)	1.01 (0.91–1.13)
Group				
Control	Ref	Ref	Ref	Ref
Intervention	0.99 (0.91–1.08)	0.98 (0.87–1.11)	1.16 (1.02–1.32)	1.02 (0.91–1.13)
Interaction				
Round * group	0.94 (0.83–1.07)	0.91 (0.76–1.09)	0.71 (0.58–0.87)	0.83 (0.71–0.97)
n	2,533	2,533	1,190	1,707

Notes: Incidence rate ratios (IRRs) and 95% confidence intervals are from Poisson regression models estimating the rate of incorrect responses to vaccine knowledge questions. Models include indicators for intervention group (control/intervention), round (pre-/post-intervention), and their interaction. The interaction term represents a difference-in-differences estimate comparing changes in incorrect responses from pre- to post-intervention between intervention and control groups. Lower IRRs indicate fewer incorrect responses (i.e., higher knowledge).

### Attitude outcomes

At the time of the pre-intervention survey, attitudes toward all four adult vaccinations were overall positive, with means between 4.05 and 5.30 (out of 6, before z-standardization), and were universally more positive at post-intervention, with means between 4.15 and 5.31. Results of linear regression modeling (Table 4) indicated that RSV attitude was statistically significantly higher in both groups at post-intervention, and although the pre-post difference was larger in the intervention group compared to the control group, this difference was not statistically significant at p < 0.05. For influenza, COVID-19, and shingles vaccines, attitude measures were not statistically significantly different from pre- to post-intervention surveys, nor when comparing pre-post differences between intervention and control groups.

**Table 4:** Difference-in-differences OLS regression results for vaccine attitude outcomes: Coefficients (CIs) for Attitude Scale.

	Influenza	COVID-19	RSV	Shingles
Round				
Pre-intervention	Ref	Ref	Ref	Ref
Post-intervention	0.066 (−0.09–0.22)	0.053 (−0.09–0.20)	0.559 (0.40–0.71)	0.051 (−0.13–0.24)
Group				
Control	Ref	Ref	Ref	Ref
Intervention	0.008 (−0.14–0.16)	0.048 (−0.10–0.19)	−0.023 (−0.17–0.12)	−0.078 (−0.26–0.10)
Interaction:				
Round * Group	−0.006 (−0.22–0.21)	−0.039 (−0.25–0.17)	0.193 (−0.02–0.41)	0.117 (−0.14–0.38)
n	1,359	1,422	1,171	906

Notes: Coefficients and 95% confidence intervals are from ordinary least squares (OLS) regression models estimating vaccine-specific attitude scale scores. Models include indicators for intervention group (control/intervention), round (pre-/post-intervention), and their interaction.The interaction term represents a difference-in-differences estimate comparing changes in attitudes from pre- to post-intervention between intervention and control groups. Attitude scores were z-standardized within each vaccine-specific distribution across both groups and time points. Higher values indicate more positive attitudes toward vaccination.

### Vaccination behaviors

At pre-intervention, vaccine coverage ranged from approximately 26% (for RSV, control group) to 75% (for influenza, control group) (Table 2, panel C). At post-intervention, vaccine rates were generally higher, ranging from 46% for RSV in the control group to 91% for influenza in the intervention group (Table 2, Panel C). At the time of the pre-intervention survey, 50% of participants had talked to a clinician about RSV vaccination. At post-intervention, 56–59% of participants had talked to a clinician about RSV vaccination (Table 2, Panel C).

Logistic regression for the behavioral outcomes (Table 5) indicated vaccination did increase statistically significantly from pre-to post-intervention for influenza, COVID-19, and RSV. Because these were unadjusted models, the predicted probabilities by group (intervention vs. control) and time (pre- vs. post-intervention survey) from this model were equivalent to the observed values reported in Table 2, panel C; modeling tested the significance of the interaction terms, shown in the “difference in differences” rows. Comparing the pre-post change across the control and intervention groups, we found a statistically significant difference in difference for influenza vaccination at p < 0.05. There was no statistically significant difference-in-difference for the other vaccination outcome measures. For influenza, there was a net 11 percentage point gain in vaccination uptake from pre- to post-intervention in the control group (i.e., 75% to 86%), and a net 22 percentage point gain in the intervention group (i.e., 69% to 91%), for a difference-in-difference of 12 percentage points.

**Table 5:** Difference-in-differences logistic regression results for vaccine behaviors: Predicted probabilities (CIs).

	Pre-Intervention	Post-intervention	Pre-Post Change
**Influenza vaccination (n = 1,148)**			
Control	0.75 (0.71–0.80)	0.86 (0.81–0.91)	+0.10*
Intervention	0.69 (0.64–0.74)	0.91 (0.87–0.95)	+0.22*
Difference-in-differences			+0.12*
**COVID-19 vaccination (n = 1,231)**			
Control	0.61 (0.57–0.66)	0.75 (0.70–0.81)	+0.14*
Intervention	0.58 (0.53–0.64)	0.77 (0.72–0.82)	+0.19*
Difference-in-differences			+0.05
**RSV Clinical Consultation (n = 1,093)**			
Control	0.51 (0.45–0.56)	0.56 (0.49–0.62)	+0.05
Intervention	0.51 (0.45–0.56)	0.59 (0.53–0.65)	+0.08
Difference-in-differences			+0.03
**RSV vaccination (n = 1,094)**			
Control	0.26 (0.21–0.30)	0.46 (0.40–0.53)	+0.20*
Intervention	0.30 (0.25–0.35)	0.51 (0.44–0.57)	+0.20*
Difference-in-differences			+0.00
**Shingles vaccination (n = 792)**			
Control	0.60 (0.53–0.66)	0.54 (0.46–0.62)	−0.06
Intervention	0.54 (0.46–0.62)	0.56 (0.48–0.64)	−0.09
Difference-in-differences			−0.03

Notes: Values are unadjusted predicted probabilities from logistic regression models including indicators for intervention group, pre/post intervention time point, and their interaction. The difference-in-differences estimate represents the differential pre–post change in vaccination coverage between the intervention and control groups. Statistical significance of the difference-in-differences effect was assessed using the p-value of the interaction term in the model.

* = significant at p=0.05 ** significant at p=0.01 *** significant at p=0.001.

In sensitivity analyses not shown, we ran models including controls for a range of demographic variables. Results had no notable differences in main effects, compared to the unadjusted models presented here. A subset analysis including only the repeat participants (also not shown) found main effects in the same direction as those reported in the main analysis. However, effects did not reach statistical significance.

## Discussion

Taken together, this study tested the impact of a subscription-based digital newsletter on study participants’ knowledge, attitudes, and behaviors related to four adult vaccinations. Results differed by vaccine. For RSV and shingles vaccines, the intervention was associated with knowledge improvement without parallel changes in attitudes and behavior. For influenza vaccine, the intervention was associated with increased vaccine uptake despite no change in knowledge or attitudes. For COVID-19, no significant effects of the intervention were observed.

Previous studies show that digital reminders and informational content can increase vaccination [22–25,28,32]. We add to this literature by showing that digital newsletters delivered via email and the Substack app/website can influence knowledge, and in some cases uptake, among self-subscribed readers, but observed effects varied by vaccine.

The intervention’s differential effects suggest that the pathway from knowledge to behavior depends on vaccine type, audience, and context. For example, influenza vaccination behavior change may have been more responsive to the intervention than other vaccines, even in the absence of change in knowledge or attitudes. We are not able to directly assess why. It is possible that because influenza vaccine knowledge was very high at baseline in our study participants, it was not likely to have increased. It is also possible that because influenza vaccine is widely available, covered by insurance, and reinforced by consistent recommendations for years, additional knowledge was not required for behavior change to occur. Overall, our additional influenza vaccine content may have functioned more as a digital reminder than informational content. Finally, our influenza vaccine content was also concentrated in November, during influenza vaccination season [43]. The timing may have amplified behavior change. By contrast, RSV vaccination content was distributed over a span of 3 months. This vaccine was also new in Fall 2023, with the CDC recommending shared decision-making rather than universal uptake. Thus providers and the public were still learning about the vaccine, and insurance coverage was uncertain. These contextual factors may explain why knowledge improved, but attitude and behavior did not, for RSV vaccination.

The theory of change underlying many vaccine promotion efforts assumes a knowledge-attitude-behavior (KAB) progression. This framework, reflected in the Health Belief Model [44] and Theory of Planned Behavior [45], suggests that knowledge informs attitude, which in turn drives behavior. Our findings, however, challenge this assumption. Given the rapid changes in the health information context, including the rise of social media and other informal sources of health information (both correct and incorrect), the relevance of these older behavioral models may be changing.

Our findings illustrate that knowledge gains are not always sufficient, or even necessary, for behavior change, even in populations for whom major access barriers are limited. For RSV and shingles, knowledge improved without attitudinal or behavioral shifts. For influenza, behavior improved without measurable changes in knowledge or attitude. These findings support critiques of knowledge-deficit approaches [46–49] and highlight the importance of taking into account the functional, emotional, social, and structural determinants of vaccination, which may modify the KAB pathway. As Brewer et al. note, thoughts and feelings matter for vaccine uptake, but so do social norms and logistical supports (e.g., free access, workplace accommodations) [47]. Knowledge change is not without value, but may not be enough to produce improvements in vaccine behavior without additional support.

### Limitations

We considered several limitations. Many effects were in the expected direction but did not reach statistical significance, suggesting the study may have been underpowered or that effects were small. Our pre-intervention survey results indicate that the study population was highly knowledgeable and had positive vaccine attitudes, so our small effects could indicate a ceiling effect on the amount of change possible in this group. The intervention “dose” or the time between intervention and follow-up may also have been insufficient, especially for newer vaccines like RSV that may require longer adoption windows or vaccines such as shingles for which there is no seasonal pattern. In addition, this study relied on repeated cross-sectional samples, not a true longitudinal panel, leaving open the possibility that differences in who responded were at play in our results. Sensitivity analyses conducted among repeat participants showed similar patterns but smaller, non-statistically significant effects, as would be expected with reduction in sample size and reduced variation due to repeated measures in a panel study design. Models adjusted for demographic factors were not substantively different in terms of main effects, supporting that the randomized assignment led to balanced assignment to control vs. intervention group. Finally, we examined multiple models, which raises the possibility of inflated Type I error due to multiple comparisons. However, the number of comparisons was limited, with analysis conducted across only 4–5 outcomes per construct (knowledge, attitude, behavior), reducing the likelihood that our findings are driven by chance alone.

It remains unclear whether changes stemmed from newsletter content specifically or from simply receiving more communications, whereby an increased number of newsletters could serve as a ‘nudge’ for behavior change. In addition, some of the intervention newsletters highlighted the timeliness and eligibility criteria for adult vaccinations, situating them more in the digital reminder space than the information space. However, average newsletter open rates were about the same across vaccines in the study (influenza 55%, COVID-19 55%, RSV 53%, shingles 54%), suggesting that differences were not due to a nudge effect alone. Also, because we did not systematically vary message framing or calls to action, we cannot identify which elements were most effective.

Finally, our sample was not representative of the general population: TNG readers broadly (i.e., across all platforms) generally have expressed high trust in its content [50]. Most participants were politically progressive, highly educated women, and had high vaccine knowledge and positive vaccine attitudes at baseline. In addition, our survey response proportion (about 10% with usable responses) could introduce respondent bias. These factors limit generalizability of our findings, though similar strategies may work in other trusted-audience contexts.

Despite these limitations, this study offers rare experimental evidence on digital newsletter interventions for improving adult vaccination uptake. Although scalability and cost were not evaluated in this study, digital newsletters represent a delivery modality that is widely used and low-cost, underscoring the value of further research to assess their effectiveness across diverse populations and contexts.

## Conclusions

Subscriber-based digital newsletters are a low-cost, scalable, and timely communication tool that can be tailored to specific audiences. Our results suggest they can increase vaccine knowledge and behavior within engaged subscriber populations, but effects on uptake depended on vaccine type, and we speculate reasons for this variation included timing and historical context of each vaccine. Future research should test this approach in more diverse populations, vary content design (e.g., behavioral science-informed framing, calls to action), and compare digital newsletters with other communication strategies. Overall, knowledge gains did not consistently translate into behavior change, underscoring the need to pair information with strategies addressing social, emotional, and structural determinants of vaccination.

## Supporting information

S1 Appendix Digital newsletter content details. (PDF)

S2 Appendix Survey instrument. (PDF)
