# Supplementary material for: A randomized assessment of the impact of ‘Those Nerdy Girls’ newsletters on adult vaccination outcomes
Source: PLoS One. 2026 Mar 12;21(3):e0344258. doi: 10.1371/journal.pone.0344258 (PMC12981454; doi:10.1371/journal.pone.0344258)
Supplement: S1 Appendix — (PDF) [file pone.0344258.s001.pdf]

S1 Appendix. Digital Newsletter Content Details

Summary of newsletter content by theme

Content of newsletters on influenza vaccine included information about flu vaccine side effects, why an updated vaccine is needed, why a high-dose flu shot is needed for older adults, whether flu shots work (i.e., efficacy), and the morbidity and mortality of influenza.

Content of newsletters on COVID-19 vaccine included COVID-19 vaccine side effects, why an updated shot is needed, how many doses are needed, whether the vaccine works (i.e., vaccine efficacy), the burden of COVID-19, and whether there is a cost for the COVID-19 vaccine.,

Content of newsletters on RSV vaccine included information on RSV vaccine side effects, whether the vaccine works (i.e., vaccine efficacy), whether the vaccine is safe, morbidity and mortality of RSV, and whether there is a cost for the RSV vaccine.

Content of newsletters on Shingles vaccine included information on shingles vaccine side effects, who is eligible for the vaccine, who needs the vaccine, how many doses are needed, the morbidity and mortality of shingles, and whether there is a cost for the shingles vaccine.

These themes are summarized in Table S1a.

Table S1a. Content of newsletters

	Side Effects	Need for updated vaccine	Doses and eligibility	Vaccine Efficacy	Vaccine safety	Morbidity/ Mortality from virus	Cost	Specific call to action
Influenza	X	X	X	X	X	X		X
COVID-19	X	X	X	X	X	X	X	X
RSV	X	n/a	X	X	X	X	X	X
Shingles	X	X	X	X		X	X	X

All Newsletters Used in this Study

The full content of the newsletters used in this study are available online at the hyperlinks listed below. Dates of publication, target vaccine, and open rates as reported by Substack are also listed. Substack open rate data are available at the message level only and have no identifying information for specific readers. Thus, we are not able to differentiate open rates between the control and intervention groups.

Table S1b. Newsletters used in this study

Newsletter title	Date	Vaccine	Open rate
Do flu vaccines work? (archival link)	Nov 1, 2023	Influenza	55%
How common are side effects from the flu vaccine? (archival link)	Nov 5, 2023	Influenza	57%
Why do I need yet another COVID vaccine? (Archival link)	Nov 9, 2023	COVID-19	54%
How long do flu shots last? (Archival link)	Nov 12, 2023	Influenza	53%
How many people die of the flu anyway? (Archival link)	Nov 16, 2023	Influenza	55%
Why are there special flu shots for older adults? (Archival link)	Nov 19, 2023	Influenza	55%
Why should older adults be concerned about RSV? (Archival link)	Nov 22, 2023	RSV	54%
Why does the flu vaccine have to be updated every year? (Archival link)	Nov 26, 2023	Influenza	54%
What COVID-19 shot am I supposed to get this Fall? (Archival link)	Nov 29, 2023	COVID-19	52%
How effective is the new RSV vaccine for older people? (Archival link)	Dec 3, 2023	RSV	54%
How common are side effects from the shingles vaccine? (Archival link)	Dec 6, 2023	Shingles	56%
Will I have to pay for my COVID-19 vaccine? (Archival link)	Dec 10, 2023	COVID-19	52%
Do we know if the new RSV vaccines are safe? (Archival link)	Dec 13, 2023	RSV	52%
Who is eligible for the shingles vaccine? (Archival link)	Dec 17, 2023	Shingles	54%

Newsletter title	Date	Vaccine	Open rate
What are the side effects of the updated COVID vaccine? (Archival link)	Dec 21. 2023	COVID-19	56%
If I already got the 1-dose shingles vaccine, should I get the 2-dose shingles vaccine now? (Archival link)	Jan 4, 2024	Shingles	55%
What are the side effects of the RSV vaccines for older adults? (Archival link)	Jan 8, 2024	RSV	51%
Can I get shingles over and over again? (Archival link)	Jan 10, 2024	Shingles	55%
Is COVID-19 surging? (Archival link)	Jan 14, 2024	COVID-19	60%
Is the new RSV vaccine covered by insurance? (Archival link)	Jan 18, 2024	RSV	53%
I never had chickenpox as a child. Do I need the shingles vaccine? (Archival link)	Jan 21, 2024	Shingles	55%
New RSV shots would save many lives if they were adopted like flu shots (Archival link)	Jan 25, 2024	RSV	56%
Are the updated COVID vaccines preventing severe disease? (Archival link)	Jan 28, 2024	COVID-19	58%
Is the shingles vaccine covered under my insurance plan? (Archival link)	Jan 31, 2024	Shingles	49%
