# Supplementary material for: A randomized assessment of the impact of ‘Those Nerdy Girls’ newsletters on adult vaccination outcomes
Source: PLoS One. 2026 Mar 12;21(3):e0344258. doi: 10.1371/journal.pone.0344258 (PMC12981454; doi:10.1371/journal.pone.0344258)
Supplement: S2 Appendix — (PDF) [file pone.0344258.s002.pdf]

# S2 Survey Instrument

## Screener Questions

**age18** Are you at least 18 years old?
o Yes
o No

→Skip To: End of Survey IF **age18** = No

**national** location Do you live in the United States of America?
o Yes
o No

→Skip To: End of Survey IF **national** = No

---

## Consent

**consent** Study Information and Consent

University of Wisconsin - Madison
Research Participant Information and Consent Form

Study Title: 2023 Those Nerdy Girls Impact Assessment
Principal Investigator: Malia Jones (Phone: 608-262-9529) (Email: malia.jones@wisc.edu)

Description of the research
You are invited to participate in a research study about the impact of reading content from Those Nerdy Girls on your vaccination behaviors and intentions. You have been asked to participate because you have subscribed to "Those Nerdy Girls" on Substack.

The purpose of the research is to determine if reading Those Nerdy Girls changes your thoughts or behaviors around vaccinations. This study will include people who are at least 18 years old and who live in the United States. This research will be conducted via an online survey, which you can complete from your mobile device or your computer.

What will my participation involve?

If you decide to participate in this research, you will be asked to complete an online survey. You will be asked to complete another similar online survey in a few months. If you choose to take this later survey, we will link your answers from both surveys together for analysis. Your participation will last approximately 8 minutes each time you choose to participate.

Are there any risks to me?

There is a chance you will become upset or experience discomfort while you are completing the survey. We will ask questions about vaccinations, which can be a sensitive subject for some people. You may stop the survey at any time.

There is a small risk of a confidentiality breach. Please read the section below, “How will my confidentiality be protected?”, for information about how we will make sure this risk is as small as possible.

Are there any benefits to me?

We don't expect any direct benefits to you from participation in this study.

How will my confidentiality be protected?

This study is confidential. Neither your name or any other identifiable information will be published. The data will be stored electronically in a UW-Madison Box folder. Only the researchers working on this study at UW-Madison will have access to the identifiable data. We will remove all identifiers from the data as soon as we are done processing them, and store the identifiers in a separate, password-protected file. The completely anonymized data may be made publicly available as part of the publication process for reproducibility purposes.

Whom should I contact if I have questions?

You may ask any questions about the research at any time. If you have questions, concerns, or complaints, or think that participating in the research has hurt you, talk to the research team or contact the Principal Investigator Malia Jones at 608-262-9529.

If you have concerns about your rights as a research participant or have complaints about the research study or study team, call the confidential research compliance line at 1-833-652-2506. UW Staff not part of the study team will work with you to address concerns and assist in resolving any complaints.

If you decide not to participate or to withdraw from the study, you may do so without penalty.

Consent to participate

By typing your name and email address below, you indicate that you have read this consent form, had an opportunity to ask any questions about your participation in this research and voluntarily consent to participate.

If you wish to keep a copy of this information for your records, please print or save one now. If you do not want to participate, you can close this window.

{Download Study Info}

**name** Please enter your first and last name to confirm you read the study information above and that you and agree to participate in this study.

**email1** Please enter your email address.

---

# Demographics

**age** How old are you?

- o 18-29
- o 30-39
- o 40-49
- o 50-59
- o 60-69
- o 70+

**gender** What is your gender?

- ▢ Man
- ▢ Woman
- ▢ Genderqueer, gender nonconforming, or non-binary
- ▢ None of these (please tell us) ________________________________________________

**trans** Do you consider yourself to be transgender?

- ▢ Yes
- ▢ No

**race/ethnicity** What is your race and/or ethnicity? [check all that apply]

- ▢ Native American or Alaska Native
- ▢ Asian
- ▢ Subcontinental Indian or Asian Indian
- ▢ Black or African American
- ▢ Hispanic or Latino
- ▢ Native Hawaiian or other Pacific Islander
- ▢ Middle Eastern or North African
- ▢ White
- ▢ Not sure

**insurance** What type of health insurance do you have? [check all that apply]

- ▢ None

▢ Medicare or Medicaid
▢ Employer-provided or other private insurance
▢ Other ______________________________________________________
▢ Not sure

**primarycare** Do you currently have a primary healthcare provider?
o Yes
o No
o Not sure

**income** Approximately what was your total household income in 2022, before taxes?
o $30,000-$70,000
o $70,001-$120,000
o $120,001+
o Not sure
o Decline to answer

**household** How many people usually lived in your household in 2022?
o 1
o 2
o 3
o 4
o 5
o 6
o 7+
o Not sure

**education** What is the highest level of education you have achieved?
o No high school diploma or G.E.D.
o High school diploma or G.E.D.
o Some college
o 2-year degree
o 4-year degree
o Master's degree
o Terminal degree (PhD, MD, JD, etc)
o Other (specify) ______________________________________________________

**politics** In terms of your political outlook, where would you place yourself?
o Very progressive
o Progressive
o Slightly progressive
o Moderate
o Slightly conservative
o Conservative

o Very Conservative
o Not sure

**location** In what state and county do you live currently?
State [Choose from list]
County [Choose from list]

---

# Knowledge Section

## Warning: false statements ahead

**text1** *In this next section, we would like to find out how much you already know about various vaccines that are available to you. Some of the statements that follow are factual, and some are not! Please check with a clinician or pharmacist if you would like to know the correct answers.*

---

## Flu Knowledge outcomes

**text2** *Please tell us if you think each statement below is true, false, or if you are not sure.*

**flu_know1** Flu vaccines are very effective at keeping people from getting infected with the flu.
o True
o False
o Not sure

**flu_know2** The best time to get a flu shot is September-October each year.
o True
o False
o Not sure

**flu_know3** Flu vaccine side effects are usually mild.
o True
o False
o Not sure

**flu_know4** Flu vaccines need to be updated every year mainly because the vaccine effectiveness wanes or "wears off" after a while.
o True
o False
o Not sure

**flu_know5** Rarely, people do get influenza from the flu vaccine.
o True
o False
o Not sure

**flu_know6** There's a high-dose flu vaccine for older adults.
o True
o False
o Not sure

---

# COVID Knowledge outcomes (COVID-19)

**text3** *Please tell us if you think each statement below is true, false, or if you are not sure.*

**cov_know1** Only people who are over age 65 or immunocompromised are eligible for an updated COVID-19 vaccine at this time.
o True
o False
o Not sure

**cov_know2** COVID-19 vaccination cannot protect against long COVID (also called post-acute sequelae of COVID-19 infection or PASC).
o True
o False
o Not sure

**cov_know3** Since the end of the federal COVID-19 emergency, COVID-19 vaccines are not covered by insurance.
o True
o False
o Not sure

**cov_know4** Updated COVID-19 vaccines keep people out of the hospital.
o True
o False
o Not sure

**cov_know5** Updated COVID-19 vaccines have very few adverse events.
o True

o False
o Not sure

**cov_know6** The updated COVID-19 vaccine is not necessary for people who already had COVID-19.
o True
o False
o Not sure

---

# RSV Knowledge outcomes (RSV)

**text4** *Please tell us if you think each statement below is true, false, or if you are not sure.*

**rsv_know1** The RSV vaccines have been around for a long time.
o True
o False
o Not sure

**rsv_know2** CDC recommends that all adults who are at least 60 years old talk to a health provider about getting an RSV vaccination.
o True
o False
o Not sure

**rsv_know3** RSV infection can be dangerous for older adults.
o True
o False
o Not sure

**rsv_know4** The RSV vaccine comes with a high risk of a serious adverse outcome, Guillain-Barre syndrome.
o True
o False
o Not sure

**rsv_know5** RSV mostly affects school-aged children.
o True
o False
o Not sure

**rsv_know6** Medicare and private insurers will cover RSV vaccine if a clinician recommends it.

o True
o False
o Not sure

---

## Shingles Knowledge outcomes (RZV/Shingles)

**text5** *Please tell us if you think each statement below is true, false, or if you are not sure.*

**shi_know1** You cannot get shingles more than once in your life.
o True
o False
o Not sure

**shi_know2** Shingles can become chronic and even fatal, especially in older people or people with immune suppression.
o True
o False
o Not sure

**shi_know3** Shingles vaccines are available to all people age 50 and up.
o True
o False
o Not sure

**shi_know4** The 1-dose shingles vaccine and 2-dose shingles vaccine offer equal protection.
o True
o False
o Not sure

**shi_know5** Anyone who has had chicken pox could get shingles.
o True
o False
o Not sure

**shi_know6** Shingles vaccines should be updated every 5 years.
o True
o False
o Not sure

---

# Sharing & Norms Section

## Signpost Sharing & Norms

**text6** *In the next section, we'll ask questions about vaccinations and the people around you.*

---

## Flu-Sharing

**flu_share1** How confident do you feel about having a conversation with other people about flu vaccination right now?
o not at all confident
o a little confident
o somewhat confident
o confident
o very confident

**flu_share2** In the last 3 months, have you forwarded any "Those Nerdy Girls" articles about annual flu vaccinations to your close friends or family members?
o Yes
o No

**flu_share3** For these next few questions, think about a friend or family member whom you are very close to, and who is an adult.

**flu_share4** How receptive would you say this person is to discussing seasonal flu vaccines with you?
o Not at all receptive
o A little bit receptive
o Somewhat receptive
o Very receptive
o I don't know

**flu_share5** How important do you think it is for this person to get a flu vaccination?
o Not at all important
o Slightly important
o Moderately important
o Very important
o Extremely important

**flu_share6** Have you talked this person about getting a flu vaccination since July, 2023 (that is, this season)?

o Yes
o No

**flu_share7** Has this person received a flu vaccine since July, 2023 (that is, this season)?

o Yes, I know that they have.
o No, I know that they have not.
o I don't know either way.

---

# RSV-sharing

**rsv_share1** How confident do you feel about having a conversation with other people about RSV vaccination right now?

o not at all confident
o a little confident
o somewhat confident
o confident
o very confident

**rsv_share2** In the last 3 months, have you forwarded any "Those Nerdy Girls" posts or articles about RSV vaccinations to your close friends or family members?

o Yes
o No

**rsv_share3** Do you have any close friends or family members who are age 60 and above?

o Yes
o No

→Skip To: End of Block IF **rsv_share3** = No

**rsv_share4** For these next few questions, think about the friend or family member whom you're closest to, and who is at least 60 years old.

**rsv_share5** How receptive would you say this person is to discussing RSV vaccines with you?

o Not at all receptive
o A little bit receptive
o Somewhat receptive
o Very receptive
o I don't know

**rsv_share6** How important do you think it is for this person to get an RSV vaccination?

- o Not at all important
- o Slightly important
- o Moderately important
- o Very important
- o Extremely important

**rsv_share7** Have you talked this person about getting an RSV vaccination?
- o Yes
- o No

**rsv_share8** Has this person had an RSV vaccination?
- o Yes, I know that they have.
- o No, I know that they have not.
- o I don't know either way.

---

# RSV Section (60+)

## Signpost 1 for Vax Outcomes/Attitudes - RSV+1

**text7** *Next we'll ask you some questions about respiratory syncytial virus (usually called RSV) and vaccines that protect against RSV. After that, we'll ask you the same set of questions for one other vaccine. We're just telling you so you know what to expect!*

---

## RSV vaccine behavioral outcomes

**rsv_convo?** Have you talked to a doctor, pharmacist, or other clinician about getting a vaccine against RSV or respiratory syncytial virus? This vaccine's brand names include Abrysvo and Arexvy.
- o Yes
- o No
- o Not sure

→ Display This Question: IF

**rsv_convo?** = Yes

**rsv_rec?** Did your doctor, pharmacist, or other clinician recommend that you should get the RSV vaccine at this time?
- o Yes
- o No

o Not sure

→ Display This Question: IF

**rsv_convo?** = Yes OR

**rsv_convo?** = Not sure

**rsv_convo_intent** Do you plan on talking to a doctor, pharmacist, or other clinician about RSV vaccine in the next 30 days?

o Yes

o No

o Not sure

**rsv_vax?** Have you ever received an RSV (respiratory syncytial virus) vaccine?

o Yes

o No

o Not sure

→Display This Question: IF

**rsv_vax?** = Yes

**rsv_vax_when?** When did you receive the RSV vaccine? You don't need to be exact here; just make your best guess.

o June 2023

o July 2023

o August 2023

o September 2023

o October 2023

o November 2023

o December 2023

o January 2024

o February 2024

o Not sure

→Display This Question: IF

**rsv_vax?** = Yes OR

**rsv_vax?** = Not sure

**rsv_vax_intent** Do you plan on getting an RSV vaccine in the next 30 days?

o Yes

o No

o Not sure

---

# RSV Attitudinal outcomes (RSV)

**text8** *To what extent do you agree or disagree with the following statements about RSV or the RSV vaccines? If you don't know, you can select "no opinion."*

## Confidence questions

**rsv_conf1** I trust that the RSV vaccines were thoroughly tested for safety in humans.
o Strongly disagree
o Somewhat disagree
o No opinion
o Somewhat agree
o Strongly agree

**rsv_conf2** I believe that FDA and the CDC made their best effort to weigh the risks and benefits of taking the RSV vaccine before recommending it.
o Strongly disagree
o Somewhat disagree
o No opinion
o Somewhat agree
o Strongly agree

**rsv_conf3** I think that we just don't know enough about the RSV vaccines.
o Strongly disagree
o Somewhat disagree
o No opinion
o Somewhat agree
o Strongly agree

**rsv_conf4** I worry about unknown future side effects of the RSV vaccines.
o Strongly disagree
o Somewhat disagree
o No opinion
o Somewhat agree
o Strongly agree

---

## Complacency questions

**rsv_comp1** I think that getting the RSV vaccine is really important for me.
o Strongly disagree
o Somewhat disagree
o No opinion
o Somewhat agree
o Strongly agree

**rsv_comp2** I believe that getting sick with RSV could have a major negative impact on my health.

- Strongly disagree
- Somewhat disagree
- No opinion
- Somewhat agree
- Strongly agree

**rsv_comp3** I worry a lot about getting sick with RSV.
- Strongly disagree
- Somewhat disagree
- No opinion
- Somewhat agree
- Strongly agree

**rsv_comp4** I already had RSV, so I don't feel like I need a vaccine for it.
- Strongly disagree
- Somewhat disagree
- No opinion
- Somewhat agree
- Strongly agree
- Not applicable: I've never had RSV

**rsv_comp5** I believe that getting sick with RSV provides immunity that is just as good as a vaccination, if not better.
- Strongly disagree
- Somewhat disagree
- No opinion
- Somewhat agree
- Strongly agree

**rsv_comp6** I think RSV is a serious threat to public health.
- Strongly disagree
- Somewhat disagree
- No opinion
- Somewhat agree
- Strongly agree

---

## Calculation questions

**rsv_calc1** I think getting the RSV vaccine will prevent me from getting sick.
- Strongly disagree
- Somewhat disagree
- No opinion

o Somewhat agree
o Strongly agree

**rsv_calc2** I think that the RSV vaccine could have serious side effects.
o Strongly disagree
o Somewhat disagree
o No opinion
o Somewhat agree
o Strongly agree

**rsv_calc3** I worry a lot about RSV vaccine side effects.
o Strongly disagree
o Somewhat disagree
o No opinion
o Somewhat agree
o Strongly agree

**rsv_calc4** I believe it's possible I could get sick with RSV this season.
o Strongly disagree
o Somewhat disagree
o No opinion
o Somewhat agree
o Strongly agree

**rsv_calc5** I think that an RSV vaccine could give me RSV.
o Strongly disagree
o Somewhat disagree
o No opinion
o Somewhat agree
o Strongly agree

**rsv_calc6** I don't have time to get sick with RSV.
o Strongly disagree
o Somewhat disagree
o No opinion
o Somewhat agree
o Strongly agree

**rsv_calc7** I don't have time to get an RSV vaccine.
o Strongly disagree
o Somewhat disagree
o No opinion
o Somewhat agree
o Strongly agree

**rsv_calc8** I don't have time to deal with the side effects of an RSV vaccine.
o Strongly disagree
o Somewhat disagree
o No opinion
o Somewhat agree
o Strongly agree

---

## Constraint questions

**rsv_const1** Getting an RSV vaccine costs too much.
o Strongly disagree
o Somewhat disagree
o No opinion
o Somewhat agree
o Strongly agree

→ Display This Question: IF
**insurance** != None
**rsv_const2** RSV vaccines are covered under my health insurance plan.
o Strongly disagree
o Somewhat disagree
o No opinion
o Somewhat agree
o Strongly agree

**rsv_const3** It’s hard to schedule an appointment to get an RSV vaccine.
o Strongly disagree
o Somewhat disagree
o No opinion
o Somewhat agree
o Strongly agree

**rsv_const4** I can get an RSV vaccine at a pharmacy or clinic near me.
o Strongly disagree
o Somewhat disagree
o No opinion
o Somewhat agree
o Strongly agree

**rsv_const5** If I get an RSV vaccine, my friends and family will question my values.

- Strongly disagree
- Somewhat disagree
- No opinion
- Somewhat agree
- Strongly agree

**rsv_const6** Getting an RSV vaccine violates my own personal values.
- Strongly disagree
- Somewhat disagree
- No opinion
- Somewhat agree
- Strongly agree

**rsv_const7** Getting an RSV vaccine is consistent with my religious beliefs.
- Strongly disagree
- Somewhat disagree
- No opinion
- Somewhat agree
- Strongly agree

---

## Collective responsibility questions

**rsv_coll1** I believe that by getting the RSV vaccine, I can protect other people around me.
- Strongly disagree
- Somewhat disagree
- No opinion
- Somewhat agree
- Strongly agree

**rsv_coll2** I think it's my responsibility to my community to get vaccinated against RSV.
- Strongly disagree
- Somewhat disagree
- No opinion
- Somewhat agree
- Strongly agree

**rsv_coll3** I think getting vaccinated against RSV is a personal choice that has little to do with other people.
- Strongly disagree
- Somewhat disagree
- No opinion
- Somewhat agree
- Strongly agree

# RSV Diffusion of Innovation (RSV)

**text9** *To what extent does each of these statements about RSV vaccines describe how you feel?*

**rsv_doi1** I think getting the RSV vaccine is better than getting sick with RSV.
o Does not describe my feelings
o Slightly describes my feelings
o Moderately describes my feelings
o Mostly describes my feelings
o Clearly describes my feelings
o No opinion

→ Display This Question: IF
**rsv_vax?** = No OR
**rsv_vax?** = Not sure
**rsv_doi2_notvaxxed** One of my worries about getting an RSV vaccine is that I might regret my decision later.
o Does not describe my feelings
o Slightly describes my feelings
o Moderately describes my feelings
o Mostly describes my feelings
o Clearly describes my feelings
o No opinion

→Display This Question: IF
**rsv_vax?** = Yes
**rsv_doi2_vaxxed** One of my worries about getting an RSV vaccine was that I might regret my decision later.
o Does not describe my feelings
o Slightly describes my feelings
o Moderately describes my feelings
o Mostly describes my feelings
o Clearly describes my feelings
o No opinion

→Display This Question: IF
**rsv_vax?** = No OR
**rsv_vax?** = Not sure
**rsv_doi3_notvaxxed** Before I make a decision either way about getting an RSV vaccine, I want to talk someone who has gotten the vaccine themselves.

o Does not describe my feelings
o Slightly describes my feelings
o Moderately describes my feelings
o Mostly describes my feelings
o Clearly describes my feelings
o No opinion

→ Display This Question: IF
**rsv_vax?** = Yes
**rsv_doi3_vaxxed** Before I made the decision to get an RSV vaccine, I wanted to talk someone who has gotten the vaccine themselves.
o Does not describe my feelings
o Slightly describes my feelings
o Moderately describes my feelings
o Mostly describes my feelings
o Clearly describes my feelings
o No opinion

**rsv_doi4** Getting the RSV vaccine is compatible with who I am as a person.
o Does not describe my feelings
o Slightly describes my feelings
o Moderately describes my feelings
o Mostly describes my feelings
o Clearly describes my feelings
o No opinion

**rsv_doi5** It's difficult to understand all the information out there about the RSV vaccine.
o Does not describe my feelings
o Slightly describes my feelings
o Moderately describes my feelings
o Mostly describes my feelings
o Clearly describes my feelings
o No opinion

**rsv_doi6** Getting the RSV vaccine is a hassle.
o Does not describe my feelings
o Slightly describes my feelings
o Moderately describes my feelings
o Mostly describes my feelings
o Clearly describes my feelings
o No opinion

# RSV Perceived norms (RSV)

**rsv_norms1** Thinking about your close friends who are at least 60 years old, how many would you guess have ever had an RSV vaccine?
- o Nearly none of them
- o A few of them
- o Some, but far from all
- o Most of them
- o Just about all of them
- o Not applicable: I have no close friends who are at least 60 years old.

**rsv_norms2** Thinking about your acquaintances who are at least 60 years old, how many would you guess have ever had an RSV vaccine?
- o Nearly none of them
- o A few of them
- o Some, but far from all
- o Most of them
- o Just about all of them
- o Not applicable: I have no acquaintances who are at least 60 years old.

**rsv_norms3** Thinking about your coworkers who are at least 60 years old, how many would you guess have ever had an RSV vaccine?
- o Nearly none of them
- o A few of them
- o Some, but far from all
- o Most of them
- o Just about all of them
- o Not applicable: I have no coworkers, or none who are at least 60 years old.

**rsv_norms4** Thinking about your family members who are at least 60 years old, how many would you guess have ever had an RSV vaccine?
- o Nearly none of them
- o A few of them
- o Some, but far from all
- o Most of them
- o Just about all of them
- o Not applicable: I have no family members who are at least 60 years old.

**rsv_norms5** In general, how many of the people who are at least 60 and who live in your area would you guess have ever had a vaccine against RSV?
- o Nearly none of them
- o A few of them
- o Some, but far from all

o Most of them
o Just about all of them

---

# Shingles Section (50+)

## Signpost 2 Shingles

**text10** *Here comes the second set of questions. We'll ask about shingles and the shingles vaccines. You're almost done!*

---

## Shingles vax outcomes (50+)

**shi_vax_ever?** Have you ever received a shingles vaccine? This vaccine is also called RZV vaccine, and brand names include Shingrix and Zostavax.
o Yes
o No
o Not sure

→ Display This Question: IF
**shi_vax_ever?** = Yes
**shi_vaxxed_which** Did the vaccine you received involve one or two doses?
o One dose (called Zostavax)
o Two doses (called Shingrix)
o Not sure

→Display This Question: IF
**shi_vax_ever?** = Yes
**shi_vaxxed_when** About when did you receive the shingles vaccine?
o In the last 5 years
o Between 5 and 10 years ago
o More than 10 years ago
o Not sure

→Display This Question: IF

**shi_vax_ever?** = No OR
**shi_vax_ever?** = Not sure

**shi_intent_30days** Do you plan on getting a shingles vaccine in the next 30 days?
o Yes
o No
o Not sure

---

# Shingles Attitudinal outcomes (Shingles)

**text11** *To what extent do you agree or disagree with the following statements about shingles and the shingles vaccine? If you don't know, you can select "no opinion."*

## Confidence questions

**shi_conf1** I trust that the shingles vaccine was thoroughly tested for safety in humans.
o Strongly disagree
o Somewhat disagree
o No opinion
o Somewhat agree
o Strongly agree

**shi_conf2** I believe that FDA and the CDC made their best effort to weigh the risks and benefits of taking the shingles vaccine before recommending it.
o Strongly disagree
o Somewhat disagree
o No opinion
o Somewhat agree
o Strongly agree

**shi_conf3** I think that we just don’t know enough about the shingles vaccine.
o Strongly disagree
o Somewhat disagree
o No opinion
o Somewhat agree
o Strongly agree

**shi_conf4** I worry about unknown future side effects of the shingles vaccine.
o Strongly disagree
o Somewhat disagree
o No opinion
o Somewhat agree

o Strongly agree

---

## Complacency questions

**shi_comp1** I think that getting a shingles vaccine is really important for me.
o Strongly disagree
o Somewhat disagree
o No opinion
o Somewhat agree
o Strongly agree

**shi_comp2** I believe that getting sick with shingles could have a major negative impact on my health.
o Strongly disagree
o Somewhat disagree
o No opinion
o Somewhat agree
o Strongly agree

**shi_comp3** I worry a lot about getting sick with shingles.
o Strongly disagree
o Somewhat disagree
o No opinion
o Somewhat agree
o Strongly agree

**shi_comp4** I already had shingles, so I don't feel like I need a vaccine for it.
o Strongly disagree
o Somewhat disagree
o No opinion
o Somewhat agree
o Strongly agree
o Not applicable: I've never had shingles

**shi_comp5** I believe that getting sick with shingles provides immunity that is just as good as a vaccination, if not better.
o Strongly disagree
o Somewhat disagree
o No opinion
o Somewhat agree
o Strongly agree

**shi_comp6** I think shingles is a serious threat to public health.

o Strongly disagree
o Somewhat disagree
o No opinion
o Somewhat agree
o Strongly agree

**shi_comp7** I never had the chicken pox, so I don't need a shingles vaccine.
o Strongly disagree
o Somewhat disagree
o No opinion
o Somewhat agree
o Strongly agree

---

## Calculation questions

**shi_calc1** I think the shingles vaccine will prevent me from getting sick.
o Strongly disagree
o Somewhat disagree
o No opinion
o Somewhat agree
o Strongly agree

**shi_calc2** I think that the shingles vaccine could have serious side effects.
o Strongly disagree
o Somewhat disagree
o No opinion
o Somewhat agree
o Strongly agree

**shi_calc3** I worry a lot about shingles vaccine side effects.
o Strongly disagree
o Somewhat disagree
o No opinion
o Somewhat agree
o Strongly agree

**shi_calc4** I believe it's possible I could get shingles at some point this year.
o Strongly disagree (1)
o Somewhat disagree (2)
o No opinion (3)
o Somewhat agree (4)
o Strongly agree (5)

**shi_calc5** I worry that the shingles vaccine could give me shingles.
- o Strongly disagree
- o Somewhat disagree
- o No opinion
- o Somewhat agree
- o Strongly agree

**shi_calc6** I don’t have time to get sick with shingles.
- o Strongly disagree
- o Somewhat disagree
- o No opinion
- o Somewhat agree
- o Strongly agree

**shi_calc7** I don’t have time to get a shingles vaccine.
- o Strongly disagree
- o Somewhat disagree
- o No opinion
- o Somewhat agree
- o Strongly agree

**shi_calc8** I don’t have time to deal with the side effects of a shingles vaccine.
- o Strongly disagree
- o Somewhat disagree
- o No opinion
- o Somewhat agree
- o Strongly agree

---

## Constraint questions

**shi_const1** Getting a shingles vaccine costs too much.
- o Strongly disagree
- o Somewhat disagree
- o No opinion
- o Somewhat agree
- o Strongly agree

→ Display This Question: IF

**insurance** != None

**shi_const2** The shingles vaccine is covered under my health insurance plan.
- o Strongly disagree
- o Somewhat disagree

- No opinion
- Somewhat agree
- Strongly agree
- Not applicable: I don't have health insurance.

**shi_const3** It's hard to schedule an appointment to get a shingles vaccine.
- Strongly disagree
- Somewhat disagree
- No opinion
- Somewhat agree
- Strongly agree

**shi_const4** I can get a shingles vaccine at a pharmacy or clinic near me.
- Strongly disagree
- Somewhat disagree
- No opinion
- Somewhat agree
- Strongly agree

**shi_const5** If I get a shingles vaccine, my friends and family will question my values.
- Strongly disagree
- Somewhat disagree
- No opinion
- Somewhat agree
- Strongly agree

**shi_const6** Getting the shingles vaccine violates my own personal values.
- Strongly disagree
- Somewhat disagree
- No opinion
- Somewhat agree
- Strongly agree

**shi_const7** Getting the shingles vaccine is consistent with my religious beliefs.
- Strongly disagree
- Somewhat disagree
- No opinion
- Somewhat agree
- Strongly agree

---

## Collective responsibility questions

**shi_coll1** I believe that by getting the shingles vaccine, I can protect other people around me.
o Strongly disagree
o Somewhat disagree
o No opinion
o Somewhat agree
o Strongly agree

**shi_coll2** I think it’s my responsibility to my community to get vaccinated against shingles.
o Strongly disagree
o Somewhat disagree
o No opinion
o Somewhat agree
o Strongly agree

**shi_coll3** I think getting vaccinated against shingles is a personal choice that has little to do with other people.
o Strongly disagree
o Somewhat disagree
o No opinion
o Somewhat agree
o Strongly agree

---

# Shingles Diffusion of Innovation (Shingles)

**text12** *To what extent does each of these statements about shingles vaccines describe how you feel?*

**shi_doi1** I think getting the shingles vaccine is better than getting shingles.
o Does not describe my feelings
o Slightly describes my feelings
o Moderately describes my feelings
o Mostly describes my feelings
o Clearly describes my feelings
o No opinion

→ Display This Question: IF
**shi_vax_ever?** = No OR
**shi_vax_ever?** = Not sure

**shi_doi2_notvaxxed** One of my worries about getting the shingles vaccine is that I might regret my decision later.
o Does not describe my feelings

o Slightly describes my feelings
o Moderately describes my feelings
o Mostly describes my feelings
o Clearly describes my feelings
o No opinion

→ Display This Question:
**shi_vax_ever?** = Yes
**shi_doi2_vaxxed** One of my worries about getting the shingles vaccine was that I might regret my decision later.
o Does not describe my feelings
o Slightly describes my feelings
o Moderately describes my feelings
o Mostly describes my feelings
o Clearly describes my feelings
o No opinion

→Display This Question:
**shi_vax_ever?** = No OR
**shi_vax_ever?** = Not sure
**shi_doi3_notvaxxed** Before I make a decision either way about getting a shingles vaccine, I want to talk someone who has gotten the vaccine themselves.
o Does not describe my feelings
o Slightly describes my feelings
o Moderately describes my feelings
o Mostly describes my feelings
o Clearly describes my feelings
o No opinion

→Display This Question:
**shi_vax_ever?** = Yes
**shi_doi3_vaxxed** Before I made the decision to get a shingles vaccine, I wanted to talk someone who has gotten the vaccine themselves.
o Does not describe my feelings
o Slightly describes my feelings
o Moderately describes my feelings
o Mostly describes my feelings
o Clearly describes my feelings
o No opinion

**shi_doi4** Getting the shingles vaccine is compatible with who I am as a person.
o Does not describe my feelings
o Slightly describes my feelings
o Moderately describes my feelings

o Mostly describes my feelings
o Clearly describes my feelings
o No opinion

**shi_doi5** It's hard to understanding all the information out there about shingles vaccines.
o Does not describe my feelings
o Slightly describes my feelings
o Moderately describes my feelings
o Mostly describes my feelings
o Clearly describes my feelings
o No opinion

**shi_doi6** Getting the shingles vaccine is a hassle.
o Does not describe my feelings
o Slightly describes my feelings
o Moderately describes my feelings
o Mostly describes my feelings
o Clearly describes my feelings
o No opinion

---

## Shingles Perceived norms (Shingles)

**shi_norms1** Thinking about your close friends who are at least 50 years old, how many would you guess have ever had a shingles vaccine?
o Nearly none of them
o A few of them
o Some, but far from all
o Most of them
o Just about all of them
o Not applicable: I have no close friends who are at least 50 years old.

**shi_norms2** Thinking about your acquaintances who are at least 50 years old, how many would you guess have ever had a shingles vaccine?
o Nearly none of them
o A few of them
o Some, but far from all
o Most of them
o Just about all of them
o Not applicable: I have no acquaintances who are at least 50 years old.

**shi_norms3** Thinking about your coworkers who are at least 50 years old, how many would you guess have ever had a shingles vaccine?
- o Nearly none of them
- o A few of them
- o Some, but far from all
- o Most of them
- o Just about all of them
- o Not applicable: I have no coworkers, or none who are at least 50 years old.

**shi_norms4** Thinking about your family members who are at least 50 years old, how many would you guess have ever had a shingles vaccine?
- o Nearly none of them
- o A few of them
- o Some, but far from all
- o Most of them
- o Just about all of them)
- o Not applicable: I have no family members who are at least 50 years old.

**shi_norms5** In general, how many of the people who are at least 50 and who live in your area would you guess have ever had a vaccine against shingles?
- o Nearly none of them
- o A few of them
- o Some, but far from all
- o Most of them
- o Just about all of them

---

# Flu vaccine section

## Signpost 2 Flu

**text13** *Here comes the second set of questions. We'll ask about seasonal flu (influenza) and the flu vaccine. You're almost done!*

---

## Flu vax outcomes

**flu_vax_lastyear** Did you get a flu vaccine (also called a flu shot) any time between July 2022 and March 2023? (That's last season.)

o Yes
o No
o Not sure

**flu_vax_thisyear** Since July 1st, 2023, have you received a flu vaccine? (That's this season.)
o Yes
o No
o Not sure

→ Display This Question: IF
**flu_vax_thisyear** = No
**flu_vax_intent_30day** Do you plan on getting a flu vaccine in the next 30 days?
o Yes
o No
o Not sure

---

# Flu Attitudinal outcomes (flu)

**text14** *To what extent do you agree or disagree with the following statements about the flu and the flu vaccine? If you don't know, you can select "no opinion."*

---

## Confidence questions

**flu_conf1** I trust that the seasonal flu vaccine was thoroughly tested for safety in humans.
o Strongly disagree
o Somewhat disagree
o No opinion
o Somewhat agree
o Strongly agree

**flu_conf2** I believe that FDA and the CDC made their best effort to weigh the risks and benefits of taking the flu vaccine before recommending it.
o Strongly disagree
o Somewhat disagree
o No opinion
o Somewhat agree
o Strongly agree

**flu_conf3** I think that we just don’t know enough about the seasonal flu vaccine.
o Strongly disagree
o Somewhat disagree
o No opinion
o Somewhat agree
o Strongly agree

**flu_conf4** I worry about unknown future side effects of the flu vaccine.
o Strongly disagree
o Somewhat disagree
o No opinion
o Somewhat agree
o Strongly agree

---

## Complacency questions

**flu_comp1** I think that getting the annual flu vaccine is really important for me.
o Strongly disagree
o Somewhat disagree
o No opinion
o Somewhat agree
o Strongly agree

**flu_comp2** I believe that getting sick with the flu could have a major negative impact on my health.
o Strongly disagree
o Somewhat disagree
o No opinion
o Somewhat agree
o Strongly agree

**flu_comp3** I worry a lot about getting sick with the flu.
o Strongly disagree
o Somewhat disagree
o No opinion
o Somewhat agree
o Strongly agree

**flu_comp4** I already had the flu, so I don’t feel like I need a vaccine for it.
o Strongly disagree
o Somewhat disagree
o No opinion

- Somewhat agree
- Strongly agree
- Not applicable: I've never had the flu

**flu_comp5** I believe that getting sick with the flu provides immunity that is just as good as a vaccination, if not better.

- Strongly disagree
- Somewhat disagree
- No opinion
- Somewhat agree
- Strongly agree

**flu_comp6** I think the flu is a serious threat to public health.

- Strongly disagree
- Somewhat disagree
- No opinion
- Somewhat agree
- Strongly agree

**flu_comp7** I worry that if I get a flu vaccine this year, I'll have to start doing it every year.

- Strongly disagree
- Somewhat disagree
- No opinion
- Somewhat agree
- Strongly agree

---

## Calculation questions

**flu_calc9** I got a flu vaccine in a previous year, so I don't feel like I need to get another one already.

- Strongly disagree
- Somewhat disagree
- No opinion
- Somewhat agree
- Strongly agree
- Not applicable: I have never had a flu vaccine.

**flu_calc1** I think getting the flu vaccine will prevent me from getting sick.

- Strongly disagree
- Somewhat disagree
- No opinion
- Somewhat agree
- Strongly agree

**flu_calc2** I think that the flu vaccine could have serious side effects.
o Strongly disagree
o Somewhat disagree
o No opinion
o Somewhat agree
o Strongly agree

**flu_calc3** I worry a lot about flu vaccine side effects.
o Strongly disagree
o Somewhat disagree
o No opinion
o Somewhat agree
o Strongly agree

**flu_calc4** I believe it's possible I could get the flu this season.
o Strongly disagree
o Somewhat disagree
o No opinion
o Somewhat agree
o Strongly agree

**flu_calc5** I worry that the flu vaccine could give me the flu.
o Strongly disagree
o Somewhat disagree
o No opinion
o Somewhat agree
o Strongly agree

**flu_calc6** I don't have time to get sick with the flu.
o Strongly disagree
o Somewhat disagree
o No opinion
o Somewhat agree
o Strongly agree

**flu_calc7** I don't have time to get a flu vaccine.
o Strongly disagree
o Somewhat disagree
o No opinion
o Somewhat agree
o Strongly agree

**flu_calc8** I don't have time to deal with the side effects of a flu vaccine.

o Strongly disagree
o Somewhat disagree
o No opinion
o Somewhat agree
o Strongly agree

---

## Constraint questions

**flu_const1** Getting a flu vaccine costs too much.
o Strongly disagree
o Somewhat disagree
o No opinion
o Somewhat agree
o Strongly agree

→ Display This Question: IF
**insurance** != None
**flu_const2** The flu vaccine is covered under my health insurance plan.
o Strongly disagree
o Somewhat disagree
o No opinion
o Somewhat agree
o Strongly agree
o Not applicable: I don't have health insurance.

**flu_const3** It’s hard to schedule an appointment to get a flu vaccine.
o Strongly disagree
o Somewhat disagree
o No opinion
o Somewhat agree
o Strongly agree

**flu_const4** I can get the flu vaccine at a pharmacy or clinic near me.
o Strongly disagree
o Somewhat disagree
o No opinion
o Somewhat agree
o Strongly agree

**flu_const5** If I get the flu vaccine, my friends and family will question my values.
o Strongly disagree

o Somewhat disagree
o No opinion
o Somewhat agree
o Strongly agree

**flu_const6** Getting the flu vaccine violates my own personal values.
o Strongly disagree
o Somewhat disagree
o No opinion
o Somewhat agree
o Strongly agree

**flu_const7** Getting the flu vaccine is consistent with my religious beliefs.
o Strongly disagree
o Somewhat disagree
o No opinion
o Somewhat agree
o Strongly agree

---

## Collective responsibility

**flu_coll1** I believe that by getting the flu vaccine, I can protect other people around me.
o Strongly disagree
o Somewhat disagree
o No opinion
o Somewhat agree
o Strongly agree

**flu_coll2** I think it's my responsibility to my community to get vaccinated against the flu.
o Strongly disagree
o Somewhat disagree
o No opinion
o Somewhat agree
o Strongly agree

**flu_coll3** I think getting vaccinated against the flu is a personal choice that has little to do with other people.
o Strongly disagree
o Somewhat disagree
o No opinion
o Somewhat agree
o Strongly agree

## Flu Diffusion of Innovation (flu)

**text15** *To what extent does each of these statements about the flu vaccine describe how you feel?*

**flu_doi1** Getting the flu vaccine is better than getting the flu.
o Does not describe my feelings
o Slightly describes my feelings
o Moderately describes my feelings
o Mostly describes my feelings
o Clearly describes my feelings
o No opinion

→ Display This Question: IF
**flu_vax_thisyear** = No OR
**flu_vax_thisyear** = Not sure
**flu_doi2_notvaxxed** One of my worries about getting a flu vaccine is that I might regret my decision later.
o Does not describe my feelings
o Slightly describes my feelings
o Moderately describes my feelings
o Mostly describes my feelings
o Clearly describes my feelings
o No opinion

→Display This Question: IF
**flu_vax_thisyear** = Yes
**flu_doi2_vaxxed** One of my worries about getting a flu vaccine was that I might regret my decision later.
o Does not describe my feelings
o Slightly describes my feelings
o Moderately describes my feelings
o Mostly describes my feelings
o Clearly describes my feelings
o No opinion

→Display This Question: IF
**flu_vax_thisyear** = No OR
**flu_vax_thisyear** = Not sure
**flu_doi3_notvaxxed** Before I make a decision either way about getting a seasonal flu vaccine, I want to talk someone who has gotten the vaccine themselves.
o Does not describe my feelings

o Slightly describes my feelings
o Moderately describes my feelings
o Mostly describes my feelings
o Clearly describes my feelings
o No opinion

→ Display This Question: IF

**flu_vax_thisyear** = Yes

**flu_doi3_vaxxed** Before I made the decision to get a seasonal flu vaccine, I wanted to talk someone who has gotten the vaccine themselves.

o Does not describe my feelings
o Slightly describes my feelings
o Moderately describes my feelings
o Mostly describes my feelings
o Clearly describes my feelings
o No opinion

**flu_doi4** Getting the flu vaccine is compatible with who I am as a person.

o Does not describe my feelings
o Slightly describes my feelings
o Moderately describes my feelings
o Mostly describes my feelings
o Clearly describes my feelings
o No opinion

**flu_doi5** It is hard to understand all the information out there about the seasonal flu vaccine.

o Does not describe my feelings
o Slightly describes my feelings
o Moderately describes my feelings
o Mostly describes my feelings
o Clearly describes my feelings
o No opinion

**flu_doi6** Getting the flu vaccine is a hassle.

o Does not describe my feelings
o Slightly describes my feelings
o Moderately describes my feelings
o Mostly describes my feelings
o Clearly describes my feelings
o No opinion

---

# Flu Perceived norms (flu)

**flu_norms1** How many of your close friends would you guess had a flu vaccine LAST season (that is, Fall 2022-Spring 2023)?
- o Nearly none of them
- o A few of them
- o Some, but far from all
- o Most of them
- o Just about all of them

**flu_norms2** How many of your acquaintances would you guess had a flu vaccine LAST season (that is, Fall 2022-Spring 2023)?
- o Nearly none of them
- o A few of them
- o Some, but far from all
- o Most of them
- o Just about all of them

**flu_norms3** How many of your coworkers would you guess had a flu vaccine LAST season (that is, Fall 2022-Spring 2023)?
- o Nearly none of them
- o A few of them
- o Some, but far from all
- o Most of them
- o Just about all of them
- o Not applicable: I have no coworkers.

**flu_norms4** How many of your family members would you guess had a flu vaccine LAST season (that is, Fall 2022-Spring 2023)?
- o Nearly none of them
- o A few of them
- o Some, but far from all
- o Most of them
- o Just about all of them

**flu_norms5** In general, how many of the people who live in your area would you guess had a flu vaccine LAST season (that is, Fall 2022-Spring 2023)?
- o Nearly none of them
- o A few of them
- o Some, but far from all
- o Most of them
- o Just about all of them

# COVID vaccine section

## Signpost 2 COVID

**text16** *Here comes the second set of questions. We'll ask about COVID-19 and the COVID-19 vaccines this time. You're almost done!*

---

## COVID vax outcomes

**cov_vax_ever?** How many doses of any COVID-19 vaccination have you ever received?

o 0
o 1
o 2
o 3
o 4
o 5+
o Not sure

**cov_vax_fall23?** Since September 15th, 2023, have you received an updated COVID-19 vaccine (also called a COVID-19 booster)?

o Yes
o No
o Not sure

→ Display This Question: IF

**cov_vax_fall23?** = No

**cov_vax_fall23_inten** Do you plan on getting an updated COVID-19 vaccine (also called a COVID-19 booster) in the next 30 days?

o Yes
o No
o Not sure

**cov_inf_ever** To your knowledge, have you ever had COVID-19?

o Yes, I've had it
o I suspect I have had it, but it wasn't confirmed with a test
o No, I have not had it as far as I know

---

# COVID Attitudinal outcomes (COVID)

**text20** *To what extent do you agree or disagree with the following statements about COVID-19 and the COVID-19 vaccines available in the U.S.? If you don't know, you can select "no opinion."*

---

## Confidence questions

**cov_conf1** I trust that the COVID-19 vaccines available in the U.S. were thoroughly tested for safety in humans.

o Strongly disagree
o Somewhat disagree
o No opinion
o Somewhat agree
o Strongly agree

**cov_conf2** I believe that FDA and the CDC made their best effort to weigh the risks and benefits of taking the COVID-19 vaccines before recommending each of them.

o Strongly disagree
o Somewhat disagree
o No opinion
o Somewhat agree
o Strongly agree

**cov_conf3** I think that we just don’t know enough about the COVID-19 vaccines.

o Strongly disagree
o Somewhat disagree
o No opinion
o Somewhat agree
o Strongly agree

**cov_conf4** I worry about unknown future side effects of the COVID-19 vaccines.

o Strongly disagree
o Somewhat disagree
o No opinion
o Somewhat agree
o Strongly agree

## Complacency questions

**cov_comp1** I think that getting a COVID-19 vaccine is really important for me.
- o Strongly disagree
- o Somewhat disagree
- o No opinion
- o Somewhat agree
- o Strongly agree

**cov_comp2** I believe that getting sick with COVID-19 could have a major negative impact on my health.
- o Strongly disagree
- o Somewhat disagree
- o No opinion
- o Somewhat agree
- o Strongly agree

**cov_comp3** I worry a lot about getting sick with COVID-19.
- o Strongly disagree
- o Somewhat disagree
- o No opinion
- o Somewhat agree
- o Strongly agree

→ Display This Question: IF

**cov_inf_ever** != No, I have not had it as far as I know

**cov_comp4** I already had COVID-19, so I don't feel like I need a vaccine for it.
- o Strongly disagree
- o Somewhat disagree
- o No opinion
- o Somewhat agree
- o Strongly agree
- o Not applicable: I have never had COVID-19.

**cov_comp5** I believe that getting sick with COVID-19 provides immunity that is just as good as a vaccination, if not better.
- o Strongly disagree
- o Somewhat disagree
- o No opinion
- o Somewhat agree
- o Strongly agree

**cov_comp6** I think COVID-19 is a serious threat to public health.
o Strongly disagree
o Somewhat disagree
o No opinion
o Somewhat agree
o Strongly agree

---

## Calculation questions

**cov_calc9** I already had a COVID-19 vaccine, so I don't feel like there's much benefit in getting another one.
o Strongly disagree
o Somewhat disagree
o No opinion
o Somewhat agree
o Strongly agree
o Not Applicable: I have never had a COVID-19 vaccine.

**cov_calc1** I think a COVID-19 vaccine will prevent me from getting sick.
o Strongly disagree
o Somewhat disagree
o No opinion
o Somewhat agree
o Strongly agree

**cov_calc2** I think that COVID-19 vaccines could have serious side effects.
o Strongly disagree
o Somewhat disagree
o No opinion
o Somewhat agree
o Strongly agree

**cov_calc3** I worry a lot about COVID-19 vaccine side effects.
o Strongly disagree
o Somewhat disagree
o No opinion
o Somewhat agree
o Strongly agree

**cov_calc4** I believe it's possible I could get COVID-19 this season.
o Strongly disagree
o Somewhat disagree

- No opinion
- Somewhat agree
- Strongly agree

**cov_calc5** I worry that a COVID-19 vaccine could give me COVID-19.
- Strongly disagree
- Somewhat disagree
- No opinion
- Somewhat agree
- Strongly agree

**cov_calc6** I don’t have time to get sick with COVID-19.
- Strongly disagree
- Somewhat disagree
- No opinion
- Somewhat agree
- Strongly agree

**cov_calc7** I don’t have time to get a COVID-19 vaccine.
- Strongly disagree
- Somewhat disagree
- No opinion
- Somewhat agree
- Strongly agree

**cov_calc8** I don't have time to deal with the side effects of a COVID-19 vaccine.
- Strongly disagree
- Somewhat disagree
- No opinion
- Somewhat agree
- Strongly agree

---

## Constraint questions

**cov_const1** Getting a COVID-19 vaccine costs too much.
- Strongly disagree
- Somewhat disagree
- No opinion
- Somewhat agree
- Strongly agree

→ Display This Question: IF

Insurance != None

**cov_const2** COVID-19 vaccines are covered under my health insurance plan.
- o Strongly disagree
- o Somewhat disagree
- o No opinion
- o Somewhat agree
- o Strongly agree
- o Not applicable: I don't have health insurance

**cov_const3** It’s hard to schedule an appointment to get a COVID-19 vaccine.
- o Strongly disagree
- o Somewhat disagree
- o No opinion
- o Somewhat agree
- o Strongly agree

**cov_const4** I can get a COVID-19 vaccine at a pharmacy or clinic near me.
- o Strongly disagree
- o Somewhat disagree
- o No opinion
- o Somewhat agree
- o Strongly agree

**cov_const5** If I get a COVID-19 vaccine, my friends and family will question my values.
- o Strongly disagree
- o Somewhat disagree
- o No opinion
- o Somewhat agree
- o Strongly agree

**cov_const6** Getting the COVID-19 vaccine violates my own personal values.
- o Strongly disagree
- o Somewhat disagree
- o No opinion
- o Somewhat agree
- o Strongly agree

**cov_const7** Getting a COVID-19 vaccine is consistent with my religious beliefs.
- o Strongly disagree
- o Somewhat disagree
- o No opinion
- o Somewhat agree
- o Strongly agree

## Collective responsibility

**cov_coll1** I believe that by getting a COVID-19 vaccine, I can protect other people around me.
- o Strongly disagree
- o Somewhat disagree
- o No opinion
- o Somewhat agree
- o Strongly agree

**cov_coll2** I think it's my responsibility to my community to get vaccinated against COVID-19.
- o Strongly disagree
- o Somewhat disagree
- o No opinion
- o Somewhat agree
- o Strongly agree

**cov_coll3** I think getting vaccinated against COVID-19 is a personal choice that has little to do with other people.
- o Strongly disagree
- o Somewhat disagree
- o No opinion
- o Somewhat agree
- o Strongly agree

# COVID Diffusion of Innovation (COVID-19)

**text17** *To what extent does each of these statements about COVID-19 vaccines describe how you feel?*

**cov_doi1** I think getting the COVID-19 vaccine is better than getting COVID-19.
- o Does not describe my feelings
- o Slightly describes my feelings
- o Moderately describes my feelings
- o Mostly describes my feelings
- o Clearly describes my feelings
- o No opinion

→ Display This Question: IF

**cov_vax_ever?** = 0

**cov_doi2_notvaxxed** One of my worries about getting the COVID-19 vaccine is that I might regret my decision later.
o Does not describe my feelings
o Slightly describes my feelings
o Moderately describes my feelings
o Mostly describes my feelings
o Clearly describes my feelings
o No opinion

→ Display This Question: IF
**cov_vax_ever?** != 0
**cov_doi2_vaxxed** One of my worries about getting the COVID-19 vaccine was that I might regret my decision later.
o Does not describe my feelings
o Slightly describes my feelings
o Moderately describes my feelings
o Mostly describes my feelings
o Clearly describes my feelings
o No opinion

→Display This Question: IF
**cov_vax_ever?** = 0
**cov_doi3_notvaxxed** Before I make a decision either way about getting a COVID-19 vaccine, I want to talk someone who has gotten the vaccine themselves.
o Does not describe my feelings
o Slightly describes my feelings
o Moderately describes my feelings
o Mostly describes my feelings
o Clearly describes my feelings
o No opinion

→Display This Question: IF
**cov_vax_ever?** != 0
**cov_doi3_vaxxed** Before I made the decision to get a COVID-19 vaccine, I wanted to talk someone who has gotten the vaccine themselves.
o Does not describe my feelings
o Slightly describes my feelings
o Moderately describes my feelings
o Mostly describes my feelings
o Clearly describes my feelings
o No opinion

**cov_doi4** Getting the COVID-19 vaccine is compatible with who I am as a person.
o Does not describe my feelings

o Slightly describes my feelings
o Moderately describes my feelings
o Mostly describes my feelings
o Clearly describes my feelings
o No opinion

**cov_doi5** It is hard to understand all the information out there about the COVID-19 vaccines.
o Does not describe my feelings
o Slightly describes my feelings
o Moderately describes my feelings
o Mostly describes my feelings
o Clearly describes my feelings
o No opinion

**cov_doi6** Getting the COVID-19 vaccine is a hassle.
o Does not describe my feelings
o Slightly describes my feelings
o Moderately describes my feelings
o Mostly describes my feelings
o Clearly describes my feelings
o No opinion

---

# COVID Perceived Norms (COVID)

**cov_norms1** How many of your close friends would you guess have ever had a vaccine against COVID-19?
o Nearly none of them
o A few of them
o Some, but far from all
o Most of them
o Just about all of them

**cov_norms2** How many of your acquaintances would you guess have ever had a vaccine against COVID-19?
o Nearly none of them
o A few of them
o Some, but far from all
o Most of them
o Just about all of them

**cov_norms3** How many of your coworkers would you guess have ever had a vaccine against COVID-19?

o Nearly none of them
o A few of them
o Some, but far from all
o Most of them
o Just about all of them
o Not applicable: I have no coworkers.

**cov_norms4** How many of your adult family members would you guess have ever had a vaccine against COVID-19?
o Nearly none of them
o A few of them
o Some, but far from all
o Most of them
o Just about all of them

**cov_norms5** In general, how many of the adults who live in your area would you guess have ever had a vaccine against COVID-19?
o Nearly none of them
o A few of them
o Some, but far from all
o Most of them
o Just about all of them

---

# Extra signposts

## Signpost 1 for Vax O/A - Shingles + 1

**text18** *Next we'll ask you some questions about shingles and the shingles vaccines. After that, we'll ask you the same set of questions for one other vaccine. We're just telling you so you know what to expect!*

---

## Signpost 1 for Vax O/A - Covid/Flu

**text19** *Next we'll ask you some questions about COVID-19 and the COVID-19 vaccines available in the US. After that, we'll ask you the same set of questions for annual flu vaccine. We're just telling you so you know what to expect!*
